# Dynamic pseudo‐continuous arterial spin labeling angiography using a 3D‐radial multi‐spoke spoiled gradient‐recalled sequence

**DOI:** 10.1002/mrm.70015

**Published:** 2025-09-17

**Authors:** Andreas Petrovic, Martin Soellradl, Thomas W. Okell, Andrew Gauden, Leon Lai, Shalini A. Amukotuwa, Roland Bammer

**Affiliations:** ^1^ Department of Radiology Monash Health Clayton Victoria Australia; ^2^ Department of Radiology and Radiological Sciences Monash University Clayton Victoria Australia; ^3^ Wellcome Centre for Integrative Neuroimaging, FMRIB Division, Nuffield Department of Clinical Neurosciences University of Oxford Oxford UK; ^4^ Department of Neurosurgery Monash Health Clayton Victoria Australia

**Keywords:** 3D‐radial, angiography, DAVF, multi‐spoke, PCASL

## Abstract

**Purpose:**

Accurate identification of arterial feeders and draining veins is critical for treatment decision‐making in patients with intracranial high‐flow vascular lesions. Currently available MRI sequences lack the temporal and spatial resolution needed for this task. A novel time‐resolved pseudo‐continuous arterial spin labeling (ASL) angiography sequence with high spatial and temporal resolution was developed, and image quality metrics relevant to clinical performance were assessed.

**Methods:**

Ten volunteers and eight patients with intracranial high‐flow vascular lesions underwent a brain MRI protocol, augmented with the new sequence with multi‐spoke (1–3) readouts and dynamic, sliding‐window reconstruction. For each of the acquisitions, image quality was assessed using a 5‐point Likert scale, as well as SNR and SNR efficiency. Spatial and temporal resolution and acquisition time were compared with standard‐of‐care sequences used to assess high‐flow vascular lesions.

**Results:**

The time‐resolved angiographic sequence achieved high isotropic spatial resolution (0.68 mm^3^), comparable to time of flight (TOF) MRA, but higher than that of contrast‐enhanced (CE)‐MRA, and a higher temporal resolution (200 ms) than CE‐MRA. Multi‐spoke acquisitions demonstrated a significant increase in SNR and SNR efficiency compared to single‐spoke acquisitions while maintaining an overall high image quality rating and at a 31% reduced scan time relative to the single‐spoke variant.

**Conclusion:**

This study demonstrated the clinical feasibility of a novel time‐resolved ASL sequence using a multi‐spoke 3D‐radial readout with a vessel‐signal optimized flip angle sweep. Sufficient SNR, superior spatial and temporal resolution to CE‐MRA, and a comparable spatial resolution to TOF MRA were achieved in a clinically reasonable acquisition time.

## 1 INTRODUCTION

Intracranial arteriovenous malformations (AVM) and dural arteriovenous fistulas (DAVF), collectively referred to as high‐flow vascular lesions, are characterized by arteriovenous shunting. They can lead to life‐threatening neurological complications, such as intracranial hemorrhage.^1^, ^2^ Treatment requires accurate assessment of arterial supply and venous drainage. The latter is particularly important because it determines the risk of neurological complications.^2^, ^3^ Accurate delineation of angioarchitecture requires imaging with both high temporal and spatial resolution. Catheter‐based cerebral digital subtraction angiography (DSA), therefore, remains the reference standard for diagnosing and accurately characterizing high‐flow vascular lesions.^2^, ^4^ However, this is an invasive test that uses ionizing radiation and requires access to an interventional neuroradiology service. The latter is not available at all hospitals. In an era where equal access to care is emphasized, non‐invasive techniques for accurate diagnosis are therefore needed.

MRI is the preferred first‐line, non‐invasive imaging test for high‐flow vascular lesions because it offers different contrast mechanisms and has better diagnostic performance than CT.^5^, ^6^ Key sequences in the MRI protocol include: time of flight MRA (TOF MRA), time‐resolved contrast‐enhanced MRA (CE‐MRA), and arterial spin labelling MRA (ASL MRA).^4^, ^7^, ^8^ TOF MRA has high spatial resolution, leading to high sensitivity and specificity for detecting high‐flow vascular lesions and their arterial feeders.^7^, ^9^ However, it lacks the temporal information necessary to accurately identify shunt volume and venous drainage, which are highly relevant to treatment planning. Although dynamic CE‐MRA offers temporal information, its temporal resolution is often inadequate for identifying earlier opacification of draining veins than normal veins, the hallmark of shunting.^4^, ^7^, ^8^ It also lacks the spatial resolution needed to identify small arterial feeders. The identification of draining veins is also hampered by the “clutter” caused by the opacification of normal venous structures.

Three‐dimensional pseudo‐continuous arterial spin labeling (PCASL) perfusion imaging with a single post‐label delay (PLD) has been shown to improve sensitivity and specificity for detecting high‐flow vascular lesions.^9^ A distinct advantage of arterial spin labeling (ASL) over dynamic CE‐MRA is that venous ASL signal is highly specific for arteriovenous shunting. This is because the signal of labeled blood decays during normal capillary transit.^9^ High signal is, therefore, only seen within venous structures into which arterial blood is either shunted or subsequently flows. A single‐delay ASL sequence only provides a “snapshot” in time, and may, therefore, miss the vein into which blood is directly shunted, precluding accurate characterization of venous drainage.^10^ However, showing the sequence of filling, that is, differentiating direct cortical vein drainage from cortical vein reflux, is critical for clinical decision‐making because direct cortical vein drainage carries a much higher risk of complications. This limitation can be overcome by using a time‐resolved acquisition of the ASL bolus passage.^11^, ^12^, ^13^, ^14^, ^15^, ^16^ Unlike CE‐MRA, time‐resolved ASL relies on the magnetic labeling of blood to generate signal and does not require exogenous contrast agent administration. It is also possible to achieve higher spatial resolution and a smaller temporal footprint, which is critical for detecting and characterizing high‐flow shunting lesions, especially high volume shunts with rapid arteriovenous shunting, that contrast‐enhanced MR angiographic techniques fail to capture.

Despite these benefits, the key challenges hindering the adoption of PCASL MRA in clinical practice remain its (1) inherently low SNR; and (2) long acquisition times, particularly for high temporal and spatial resolution sequences. Various strategies have been explored to address these limitations.^12^, ^14^, ^17^, ^18^, ^19^ In terms of SNR, PCASL labeling^20^ and non‐Cartesian readouts^18^ are beneficial, as demonstrated in studies by Kopeinigg et al.^15^ and Wu et al.,^16^, ^21^ which produced high‐resolution angiograms.

In this study, we build on these methods by improving scan time and SNR, while retaining the beneficial features of the 3D radial approach.^16^, ^21^ To reduce scan time and increase SNR, we used a time‐resolved, Look‐Locker‐type, multi‐spoke 3D‐radial PCASL spoiled gradient‐recalled (SPGR) sequence with numerically optimized flip angles during the readout train. Similar to Cartesian turbo field echo‐planar ASL sequences,^22^ radial multi‐spoke readouts can substantially improve sampling efficiency, compared to single‐spoke readouts, while simultaneously increasing TR and decreasing the number of excitation pulses. As a result, larger flip angles can be used to improve SNR. Additionally, the 3D‐radial trajectory allows for high isotropic spatial resolution and high temporal resolution, with the option of a sliding‐window reconstruction.^15^ The clinical feasibility of this new sequence was assessed in healthy volunteers and patients with vascular malformations.

## 2 METHODS

**FIGURE 1:**
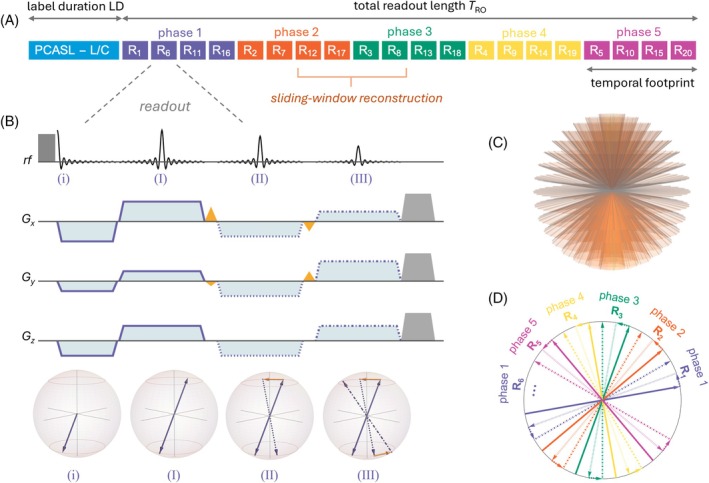
A schematic diagram of the entire sequence. (A) One shot combining the pseudo‐continuous arterial spin labeling (PCASL) module and readout train. After the labeling module, 20 readouts of duration TR are played out, constituting the total readout length (*T*_RO_), which is further subdivided into five temporal phases (*N*_p_). The total readout length and the number of temporal phases determine the temporal footprint of each time frame (*T*_RO_/*N*_p_). (B) During each readout, multiple spokes (*N*_sp_) are acquired after a single excitation (one FID and 3 spokes in this example). k‐Space traversal is depicted below the readout diagram, where radial spokes are interleaved by azimuthal blips, connecting the individual k‐space lines (see also Video [Link], [Link]). (C) The acquired spokes lie on concentric cones, with cones at smaller polar angles containing fewer spokes than those at larger angles to ensure equal k‐space sampling density. (D) A 2D view (equator) of how k‐space is covered in the azimuthal plane is shown for a 5‐phase, three‐spoke acquisition. Distinct colors represent temporal phases, whereas line styles denote individual spokes in a multi‐spoke readout. For the entire readout, the azimuthal angles are sampled in a way to generate a homogenously sampled k‐space for each phase (see readout number in [A]). This, and the radial nature of the sequence, allow for pooling of phases and sliding window reconstructions. To ensure sufficient k‐space sampling, the whole module (A) is repeated for a certain number of shots (*N*_sh_), alternating between label and control preparations.

Figure 1 outlines the overall sequence structure. The sequence consisted of the repeated application of a PCASL module followed by a multi‐spoke 3D‐radial SPGR Look‐Locker readout.

### 2.1 Labeling

For the PCASL module, Gaussian pulses (600‐μs duration, 30° flip angle, time bandwidth product = 1.8) were used in combination with 8 mT/m tagging gradients, an average gradient strength of 0.8 mT/m, and an inter‐pulse‐interval of 1.2 ms. Label and control data were acquired alternately to minimize temporal separation and motion‐related misregistration between the label and control data acquisition runs. The labeling plane was positioned at the level of the distal V2 segment of the vertebral arteries.

To achieve good shim results at the site of the labeling plane, the shim volume was manually enlarged to cover both the imaging volume and labeling plane (preliminary experiments showed that this yielded better angiographic results than shimming the imaging volume alone, most likely because of the PCASL's sensitivity to off‐resonance effects^23^).

### 2.2 Trajectory

In a 3D‐radial acquisition, the direction of radial spokes can be given by their polar and azimuthal angles. According to a given number, *n*_z_, of polar angles, θ_*i*_, the surface of the acquired k‐space sphere with radius $k_{\max}=0.5\cdot \text{Matrix}/\mathrm{FOV}$ was subdivided into *n*_z_ rings of varying size, comprising the location for endpoints of the radial spokes. For each of those rings, a certain number, *n*_***+**,i*_, of azimuthal angles, φ_*i*,*j*_, was sampled to maintain a homogeneous k‐space density across the entire sphere's surface. The polar angles were computed as follows:

(1) $$\theta_{i}=\pi \frac{i}{n_{z}}\;\text{with}\;i=0\ldots \left(n_{z}-1\right),\text{and}\;n_{z}=\text{ceil}\left(\pi \sqrt{\frac{N}{2\pi }}\;\right),$$

with *n*_z_ being the number of polar angle‐steps, and *N* being the overall number of desired spokes. From that, the azimuthal angles can be given as

(2) $$\begin{array}{cc} \varphi_{i,j} & =2\pi \frac{j}{n_{+,i}}\;\text{with}\;j=0\ldots \left(n_{+,i}-1\right),\;\text{and}\\ n_{+,i} & =\text{round}\left(\sin\left(\frac{i}{n_{z}}\pi \right)\cdot n_{z}\right)=\text{round}\left(\sin\left(\theta_{i}\right)\cdot n_{z}\right), \end{array}$$

with *n*_*+,i*_ being the number of azimuthal steps for each polar angle. With this scheme, the resulting spokes lie on concentric cones as indicated in Figure 1C. Because full echoes (from −*k*_max_ to *k*_max_) were acquired and φ varied between 0 and 2π, each spoke was covered twice, the second time in the opposite direction through k‐space.

In multi‐spoke readouts, azimuthal blip gradients were used to acquire *N*_sp_ spokes with slightly different directions through k‐space. The 0th‐order moment of the gradient blips was computed as follows:

(3) $${\vec{M}}_{\text{blip}}=2M\sin\theta \sin\left(\frac{1}{2}∆\theta \csc\theta \right)\left(\begin{array}{c} -\sin\left(\varphi +\frac{1}{2}∆\theta \csc\theta \right)\\ \cos\left(\varphi +\frac{1}{2}∆\theta \csc\theta \right)\\ 0 \end{array}\right),$$

with $∆\theta =\pi /n_{z}$ being the polar angle step, and *M* being the total 0th‐order moment of the readout gradient at the time before the blip. Such a blip moves the k‐space trajectory from the endpoints of a spoke with azimuthal angle φ to the starting points of a new spoke with an angle φ+∆φ=φ+∆θcscθ.

### 2.3 Readout

Each SPGR readout module (Figure 1B) starts with a 300‐μs hard‐pulse excitation followed by radial pre‐phasing gradients, during which a ramp‐sampled ultra‐short‐echo‐time FID is acquired. Thereafter, rephasing gradients generate an echo, traveling k‐space in the opposite direction. Optionally, instead of measuring a single echo per RF pulse, one or more (*N*_sp_‐1) azimuthal blips and readout gradients can be used to generate *N*_sp_ echoes, improving the sequence's efficiency. Finally, the remaining transverse magnetization is dephased using RF and gradient spoiling.

### 2.4 Temporal resolution

Figure 1 illustrates a single label‐control run (shot) of the sequence with *N*_p_ = 5 temporal phases (shown in different colors), *N*_ex_ = 20 RF excitations (R_1_ … R_20_), and *N*_sp_ = 3 full echoes per multi‐spoke readout (shown in different line styles). Because of the desired high spatial resolution of MR angiographic scans, the 3D k‐space requires *N*_sh_ shots until the entire k‐space is filled. After each label (or control) module, the magnetization of inflowing blood is observed with a time‐segmented Look‐Locker style readout, and multiple spokes are dynamically acquired during the readout duration (*T*_RO_). *T*_RO_ is computed as *N*_ex_ · TR, where *N*_ex_ represents the total number of RF excitation pulses in a complete Look‐Locker readout. The readout is, then, partitioned into *N*_p_ temporal phases of equal length, allowing for effective tracking of the PCASL bolus through the arterial tree.

The number of spokes, pooled together during one dynamic k‐space readout, constitutes the temporal footprint of the MR angiogram. The fewer spokes are combined, the better the temporal footprint, but the longer the total scan duration becomes.

The total scan duration is 2 · *N*_sh_ · (LD + *T*_RO_), where LD denotes the label duration. LD and *T*_RO_ together comprise the repetition time for a single Look‐Locker shot. Immediately after one shot, a new ASL preparation is applied to the neck, during which the tissue in the imaging volume relaxes back to equilibrium.

The sampling of azimuthal angles is performed in a segmented fashion, such that every *N*_*p*_‐th readout belongs to the same temporal phase (Figure 1A). Therefore, a homogenously sampled k‐space is acquired for each separate temporal phase. By carefully selecting spokes, one can share some of the radial projections for different temporal frames (i.e., view sharing), allowing for better SNR and a smoother appearance of the bolus wash‐in (i.e., sliding‐window reconstruction).

### 2.5 Flip angle optimization

To ensure good arterial contrast throughout the entire readout pulse train, it is paramount to consider the effect of repeated RF excitations and T_1_ relaxation, which drives the ASL bolus into a steady state over time. Both reduce the ASL‐signal difference and, therefore, angiographic contrast. Optimized flip angles can help maintain a constant signal difference and maximize bolus utilization. Although recursive solutions have been proposed previously^19^, ^24^ to compute the flip angle sweep, a closed‐form solution that accounts for T_1_ relaxation and the total number of excitation pulses can be given (Eq. [4]), which can readily be evaluated with the desired sequence parameters and an estimate for T_1_ of arterial blood (approx. 1600 ms^25^, ^26^). Hereby, one can calculate individual flip angles within the SPGR readout train such that the flip angle at the final RF pulse becomes 90° and, therefore, fully uses the available magnetization.

(4) $$\alpha \left[n\right]=\cos^{-1}\sqrt{1+\frac{E_{1}^{2}-1}{E_{1}^{2\left(n-K\right)}-E_{1}^{2}}}$$

here, α[*n*] is the *n*th flip angle, *K* is the number of excitations within one shot, and $E_{1}=e^{-\mathrm{TR}/T_{1}}$ with TR being defined as the time between two excitation pulses.

The optimization of flip angles was aimed at a directed modulation and full utilization of the available magnetization during the RF‐pulse train, whereby we tried to keep the difference of the inflowing blood signal between the label and control image constant for the entire RF‐pulse train. Figure S1 shows steadily increasing flip angle evolutions computed using Eq. (4).

### 2.6 Reconstruction and postprocessing

Label and control acquisitions were subtracted in k‐space, followed by coil compression using principal component analysis.^27^ k‐Space filtering was performed using a radially symmetric Welch apodization window, and sampling density correction was applied according to the squared distance from the k‐space center. Each temporal phase was reconstructed individually using an in‐house gridding algorithm. Additionally, a composite reconstruction combining all k‐space lines of the first three temporal phases was generated, thereby increasing the SNR of the angiogram. For multi‐spoke acquisitions, individual echoes as well as the additional FID were reconstructed separately and combined in magnitude mode to avoid destructive interference effects because of B_0_ inhomogeneities. A sliding‐window reconstruction increased the nominal temporal resolution to 20 ms.

As a postprocessing step, post‐ASL‐bolus inflow subtraction was performed as described by Kopeinigg et al.^15^ This method normalizes the data and subtracts each temporal phase from the first, simulating an inflowing bolus in contrast to the outflowing bolus of native PCASL MRA.

Finally, maximum intensity projections (MIPs) were computed in axial, coronal, and sagittal planes for both the *N*_p_ individual time points and the combined high‐SNR data set. Sinc‐interpolation was used to interpolate the MIPs to a matrix three times their original size.

All reconstructions were performed offline on a Windows workstation with a 12‐core Intel i7 processor (2100 MHz) and 128 GB RAM. Execution times for the multi‐threaded gridding algorithm ranged from 26 min for one‐spoke acquisitions to 48 min for three‐spoke acquisitions.

### 2.7 Experiments

Our organization's institutional review board approved the study, and all human subjects (10 healthy volunteers, 8 patients) gave written informed consent after the nature of the study was explained to them. All experiments were conducted on a 3 T scanner (Vida XQ, XA50, Siemens Healthineers) using a 64‐channel head coil.

#### 2.7.1 Volunteers

The experiments aimed to determine the feasibility of the multi‐spoke approach and whether multi‐spoke scans can be obtained without loss in image quality. To this end, 10 healthy volunteers were scanned using the proposed sequence, and three PCASL MRAs were acquired consecutively with (*N*_sp_) 1, 2, and 3 spokes per RF excitation in the readout train (Figure 1B). For each of the three acquisitions, the overall number of spokes (*N*) was kept at approximately 16 200. Using multiple spokes per RF excitation allowed for a more efficient readout, reducing the number of label/control readout passes (*N*_sh_) and significantly shortening scan time.

The scan parameters common to all acquisitions included a matrix size of 320 × 320 × 320, an isotropic resolution of 0.69 mm^3^, and a bandwidth of 280 Hz/Px. Each readout was preceded by a labeling module with LD = 2 s and no PLD (PLD = 0 s). The readout (*T*_RO_ = 1 s) was further divided into *N*_p_ = 5 phases, yielding a temporal footprint of 200 ms per phase. See Table 1 for a detailed listing of parameters. For comparative assessment, conventional multi‐slab 3D TOF MRAs were acquired in these participants.

**TABLE 1:** PCASL MRA sequence parameters for volunteers and patients.

Parameter	Volunteers and patients			Patients	
Matrix size		320		192	256
Isotropic resolution [mm^3^]		0.69		1.15	0.86
Bandwidth/pixel [Hz/Px]		280		540	220
1st TE [ms]		0.17		0.17	
TR [ms]	7.5	11.4	15.3	8.3	14.0
No. of spokes (*N*_sp_)	1	2	3	5	3
No. of reconstructed temporal phases (*N*_p_)		5		5	
No. of excitations per shot (LL readout, *N*_ex_)	135	90	65	190	100
No. of shots (LL readouts, *N*_sh_)	120	90	83	60	70
No. of spokes total (*N = N*_sh_ *· N*_ex_ *· N*_sp_)	16 200	16 200	16 185	57 000	21 000
No. of spokes per phase (*N*/*N*_p_)	3240	3240	3237	11 400	4200
Total readout length (*T*_RO_ = *N*_ex_ *·* TR) [s]	1.0	1.0	0.98	1.58	1.4
Undersampling factor	20	20	20	2	10
Temporal footprint (*T*_RO_/*N*_p_) [ms]	202	202	199	316	280
LD [s]		2		1.5	2
PLD [s]		0		0	
*T*_acq_ [min:s]	12:03	9:05	8:17	6:09	7:56

Abbreviations: LD, label duration; PCASL, pseudo‐continuous arterial spin labeling; PLD, post‐label delay; *T*_acq_, acquisition duration.

#### 2.7.2 Patients

Eight patients (5 male, 3 female, age 28–75), who had been referred to our department for the diagnostic workup of a high‐flow shunting lesion using standard diagnostic imaging (including 3D TOF‐ and CE‐MRA), were also scanned with the new PCASL MRA sequence (see Table 2). All patients underwent DSA before or after the MRI to determine whether a shunt was present and to accurately delineate venous drainage. As we started scanning patients during protocol optimization, images were acquired using three different protocols. Matrix sizes ranged from 192, 256, to 320, yielding isotropic resolutions of 1.15, 0.86, and 0.69 mm^3^, respectively. Label durations ranged from 1.5 to 2 s, with readout lengths between 1 and 1.58 s. For detailed protocol parameters, we refer to Table 1.

**TABLE 2:** Patient demographics.

No.	Age	Gender	Clinical history	Protocol
1	46	M	Cognard IV right tentorial DAVF	Patients (matrix size 192)
2	35	M	Cognard IIb superior sagittal sinus DAVF	Patients (matrix size 192)
3	28	F	Left parietotemporal AVM with superficial venous drainage	Patients (matrix size 256)
4	51	F	Cognard IIb left transverse sinus DAVF	Volunteers and patients (*N*_sp_ = 3)
5	56	M	Diploic AVF	Volunteers and patients (*N*_sp_ = 3)
6	42	M	Cognard IIa right sigmoid sinus DAVF	Volunteers and patients (*N*_sp_ = 3)
7	42	M	Left temporal AVM with superficial venous drainage	Volunteers and patients (*N*_sp_ = 3)
8	75	F	Cognard IIa right hypoglossal DAVF	Volunteers and patients (*N*_sp_ = 3)

Abbreviations: AVF, arteriovenous fistulas; AVM, arteriovenous malformations; DAVF, dural arteriovenous fistulas; F, female; M, male.

#### 2.7.3 Three‐dimensional TOF MRAs

An axial flow‐compensated (in readout and slice direction) SPGR sequence with TR = 23 ms, TE = 3.7 ms, ramped flip angle excitation (flip angle 15°, 70% ramp), and bandwidth of 186 Hz/pixel was used to acquire multi‐slab 3D TOF MRAs. Nine overlapping slabs (20% overlap, 20% slice oversampling) were scanned for high‐resolution full brain coverage, with a total acquisition time of 9:57 min. Each slab was scanned with a distally adjacent tracking saturation band (width, 40 mm; distance, 10 mm). The acquired volume had a resolution of 0.58 × 0.52 × 1 mm^3^ (i.e., FOV 192 × 200 mm^2^; slice thickness, 1 mm; acquisition matrix 348 × 384 × 148; reconstruction matrix 696 × 768 × 296; reconstructed resolution 0.26 × 0.26 × 0.5 mm^3^). In addition to partial Fourier imaging in phase (7/8) and slice (6/8) direction, parallel imaging was applied in phase encoding direction (GRAPPA 2). One patient was scanned with only five overlapping slabs, yielding a scan time of 5:06 min.

#### 2.7.4 Dynamic 3D CE‐MRA

A time‐resolved 3D SPGR sequence (TWIST) was used to scan 40 volume instances at a temporal resolution of 1.65 s in the sagittal plane. The acquired spatial resolution was 1.63 × 1.14 × 1.6 mm^3^ (i.e., FOV 256 × 256 mm^2^; slice thickness, 1.6 mm; acquisition matrix 156 × 224 × 120; reconstruction matrix 224 × 224 × 160; reconstructed resolution 1.1 × 1.1 × 1.2 mm^3^). A non‐selective RF pulse with a flip angle of 17°, TR = 2.14 ms, TE = 0.87 ms, and bandwidth of 744 Hz/pixel was used for the readout. Parallel and partial Fourier imaging were performed in phase (GRAPPA 2, PF 6/8) and slice (GRAPPA 3, PF 6/8) direction. The total acquisition duration was 1:17 min.

#### 2.7.5 DSA

DSAs were acquired with an image pixel spacing of 0.19 to 0.26 mm^2^, a matrix size of 1024 × 1024, 8 to 25 frames, and a temporal resolution ranging from 167 to 500 ms. The image dose‐area product and effective dose of a single fluorographic series were between 12 and 67 dGy · cm^2^ and 0.044 and 0.23 mSv (ICRP 103).

### 2.8 Image evaluation

For the composite PCASL MRA reconstruction of temporal phases 1 to 3, the MIPs were graded according to the overall image appearance and sharpness using a five‐point Likert scale: inferior (1), poor (2), acceptable (3), good (4), and very good (5). Additionally, the occurrence and severity of artifacts were assessed and graded using a three‐level Likert scale: none (0), mild (1), and severe (2). For every dynamic PCASL MRA series, each temporal MIP was inspected and graded for overall image quality and artifacts (Likert scales as above).

Furthermore, a quantitative SNR analysis was performed, comparing SNR across different multi‐spoke acquisitions and temporal phases. To that end, we closely followed the steps outlined in Berry et al.^18^ to compute the mean signal. The pixel values for noise estimation were taken from cubes of 30 × 30 × 30 pixels at the corners of the imaging volume. We also compared the SNR efficiency of the multi‐spoke sequence variants, as acquisition time‐normalized SNR, that is, $\mathrm{SNR}/\sqrt{T_{\mathrm{acq}}}$, where *T*_acq_ is the acquisition time.

The results of the quantitative SNR analysis were pairwise compared using t‐tests adjusted for multiple comparisons (R software package). Linear regression was used with temporal phases as covariates to assess whether image ratings were influenced by phase number.

In the eight patients, subjective assessment of visualization of feeding arteries and draining veins on ASL—compared to TOF MRA and dynamic CE‐MRA—was performed by an experienced neuroradiologist (S.A.A.) and a senior imaging scientist (R.B.) in consensus.

## 3 RESULTS

The spatial resolution of the new sequence was superior to that of CE‐MRA and only slightly inferior to that of our standard‐of‐care 3D TOF MRA. The temporal resolution was higher than that of dynamic CE‐MRA and comparable to DSA (see Table 3).

**TABLE 3:** Spatial and temporal resolution.

	Spatial resolution	Temporal resolution	Acquisition duration
DSA	0.19–0.26 mm^2^	167–500 ms	N.A.
TOF	0.58 × 0.52 × 1 mm^3^	N.A.	9:57 min
Dynamic CE‐MRA	1.63 × 1.14 × 1.6 mm^3^	1650 ms	1:17 min
PCASL MRA	1.15 mm^3^ (matrix 192)	320 ms	6:09 min
	0.86 mm^3^ (matrix 256)	280 ms	7:56 min
	0.67 mm^3^ (matrix 320)	200 ms	8:17–12:03 min

Abbreviations: CE, contrast‐enhanced; DSA, digital subtraction angiography; PCASL, pseudo‐continuous arterial spin labeling; TOF, time of flight.

### 3.1 Volunteer scans

**FIGURE 2:**
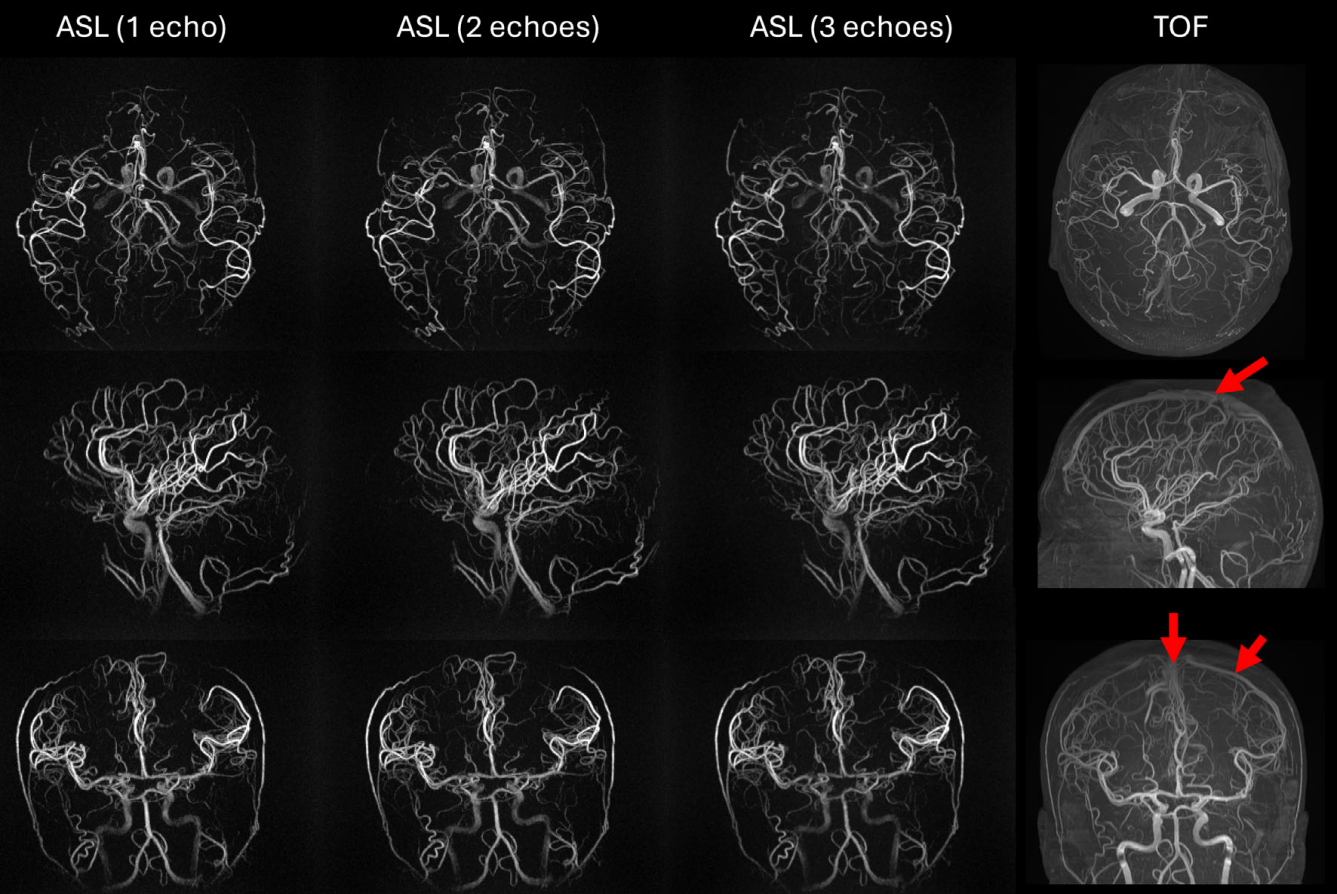
Comparison of pseudo‐continuous arterial spin labeling (PCASL) MRA with 1 to 3 spokes and time of flight (TOF) in a 35‐year‐old healthy volunteer. PCASL MRAs demonstrate excellent resolution and SNR, comparable to or better than that of TOF. TOF images show a substantial amount of venous signal (red arrows) in normal veins, whereas there is no such “venous contamination” of arterial spin labeling (ASL) images.

Figure 2 compares composite high‐SNR reconstructions (combined temporal phases 1–3) of PCASL MRA acquisitions with 1‐, 2‐, and 3‐spoke readouts (i.e., full spokes) per TR vis‐à‐vis 3D TOF MRAs (see also Figure S2). The isotropic resolution of the PCASL MRA sequence enabled multi‐planar reformatting in arbitrary directions with high spatial resolution and fine vascular detail. MIPs derived from 3D TOF MRAs display a comparable level of detail and excellent SNR, with a slightly lower through‐plane resolution. An advantage of the PCASL MRA is that there is no venous contamination, whereas substantial unsuppressed venous signal is seen on TOF MRA in the superior sagittal sinus and cortical veins because of craniocaudal flow direction and fast flow in these venous structures.

**FIGURE 3:**
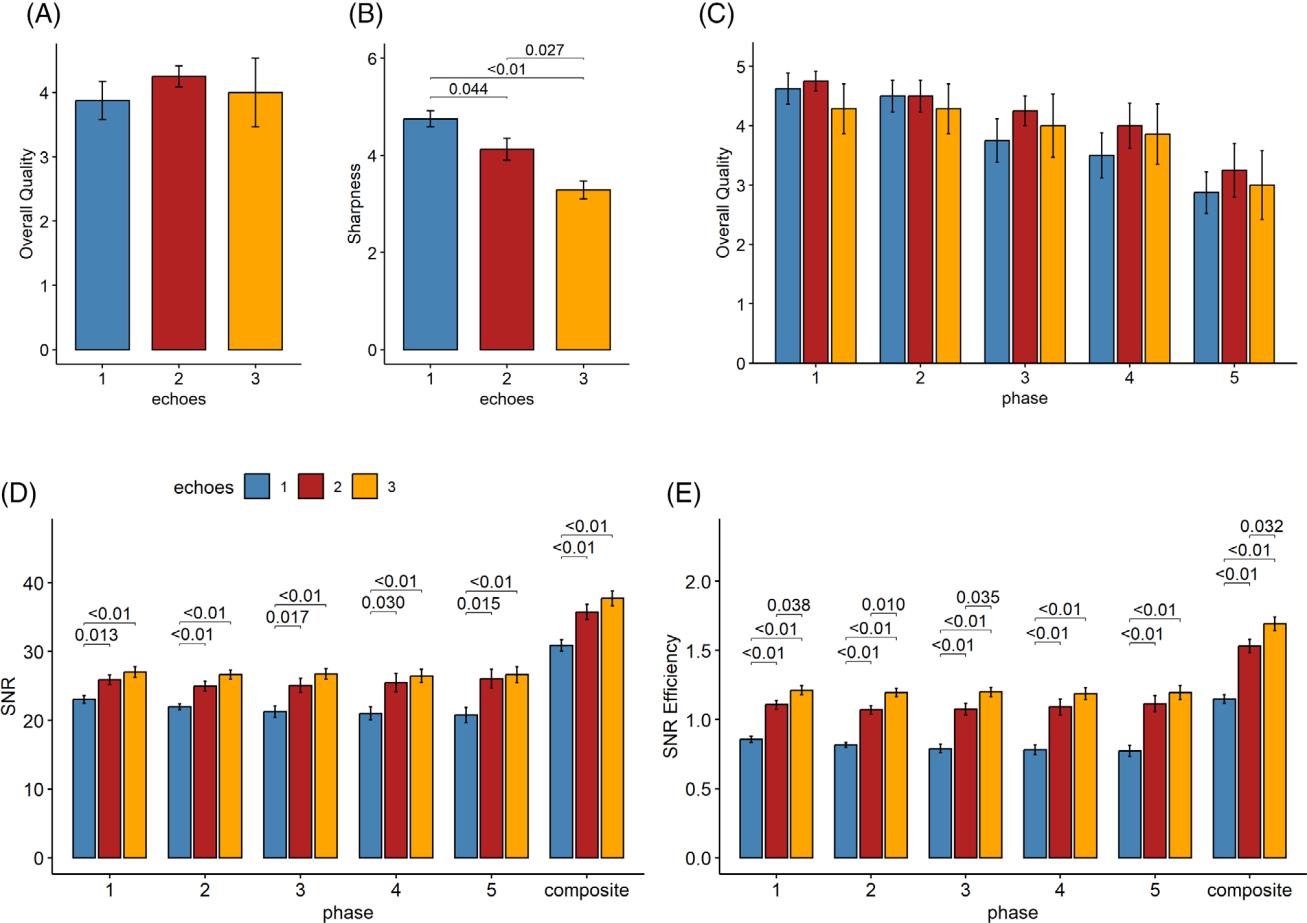
Results of the image assessment showing mean scores and standard errors, with significant differences indicated with brackets and *p*‐values, respectively. For the composite images, the overall quality (A) does not change, whereas sharpness decreases significantly (B) with the number of spokes. Looking at individual temporal phases, the overall quality ratings decrease for later phases (C). SNR values (D) and SNR efficiency (E) increased significantly for two and three‐spoke acquisitions.

Increasing the number of spokes from 1 to 3 reduced scan time by 31% (from 12:03 to 8:17 min) without significant loss in image quality (Figure 3A). Sharpness, however, decreased significantly for the multi‐spoke acquisitions (Figure 3B). Grading the quality of individual temporal phases revealed no significant differences between the sequence variants (Figure 3C). Yet there is a trend of diminishing quality for later temporal phases (decrease of −0.37 score points per phase, *p* < 0.01), with an increased likelihood of artifacts. The average artifact scores were below 0.1 for all phases except the last (0.35). In terms of the number of spokes, the 3‐spoke acquisition had a higher artifact score (0.29) compared to the other scans (<0.1). An example of increased streaking artifacts in the last phase of a 3‐spoke acquisition is shown in Figure S3. The composite MIPs contained no artifacts, except for one scan (severe).

The SNR analysis revealed a significant increase in SNR for all 3‐spoke and 2‐spoke scans across all temporal phases and the composite reconstruction, compared to the single‐spoke acquisitions (Figure 3D). The same trend can also be observed for SNR efficiency, with multi‐spoke acquisitions performing significantly better (Figure 3E).

**FIGURE 4:**
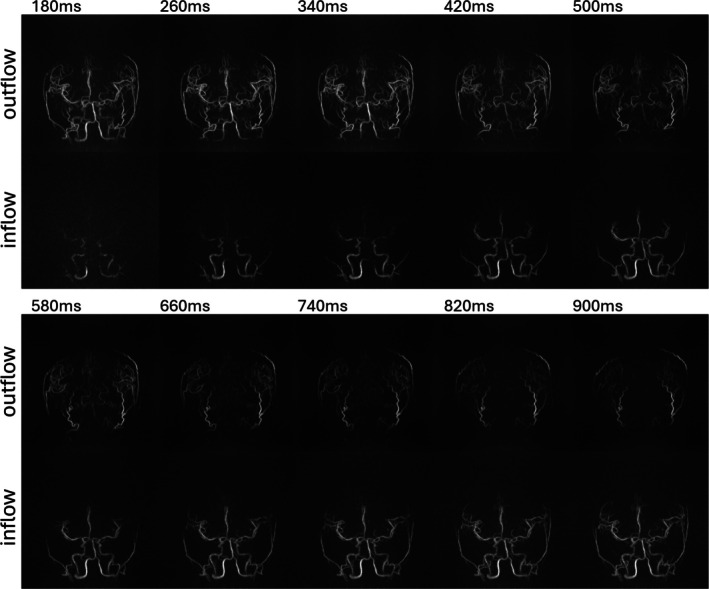
Sliding‐window reconstruction of pseudo‐continuous arterial spin labeling (PCASL) MRA scan with 3 spokes per TR readout train, allowing angiographic phase image to be reconstructed every 20 ms (temporal increment) and a temporal footprint of each angiographic phase of 200 ms in a 25‐year‐old healthy volunteer. To reduce clutter, only every fourth coronal frame is shown (i.e., a spacing of 80 ms). Both outflow and inflow maximum intensity projections (MIPs) are displayed one above the other for comparison. Although the overall information content is similar in both reconstructions, inflow MIPs provide a more natural angiographic appearance, resembling the early and mid‐arterial phases of contrast‐enhanced (CE)‐MRA, but with superior spatial resolution.

Figure 4 shows PCASL MRAs generated using a sliding‐window reconstruction and compares the native outflow contrast to the post‐processed inflow subtraction. Whereas the outflow contrast depicts more details for distal vessel segments, the inflow‐subtracted images provide a more familiar appearance of a contrast bolus filling the arterial system. The five full k‐space datasets acquired over the Look‐Locker‐readout period afforded view‐sharing to create 41 temporal frames with a 200‐ms temporal footprint and a temporal resolution of 20 ms. This figure presents only 10 selected frames to avoid clutter, showing an image every 80 ms. Time series with this enhanced temporal resolution enabled the creation of videos visualizing arterial dynamics (see Supporting information), avoiding jerky motion of the bolus as it progresses through the vascular system.

### 3.2 Patient scans

Visualization of feeding arteries on ASL was superior to that on dynamic CE‐MRA and equivalent to TOF MRA for all eight patients. In seven cases, ASL signal was identified in venous structures, which were confirmed to be draining veins on DSA. In one case, no venous ASL signal was identified. DSA 3 weeks earlier confirmed spontaneous resolution of the intraosseous arteriovenous fistula (AVF) identified on MRI a few months earlier. Shunting was, therefore, identified with 100% sensitivity and specificity on the new ASL sequence. Visualization of draining veins on ASL was superior to that on TOF MRA and dynamic CE‐MRA.

**FIGURE 5:**
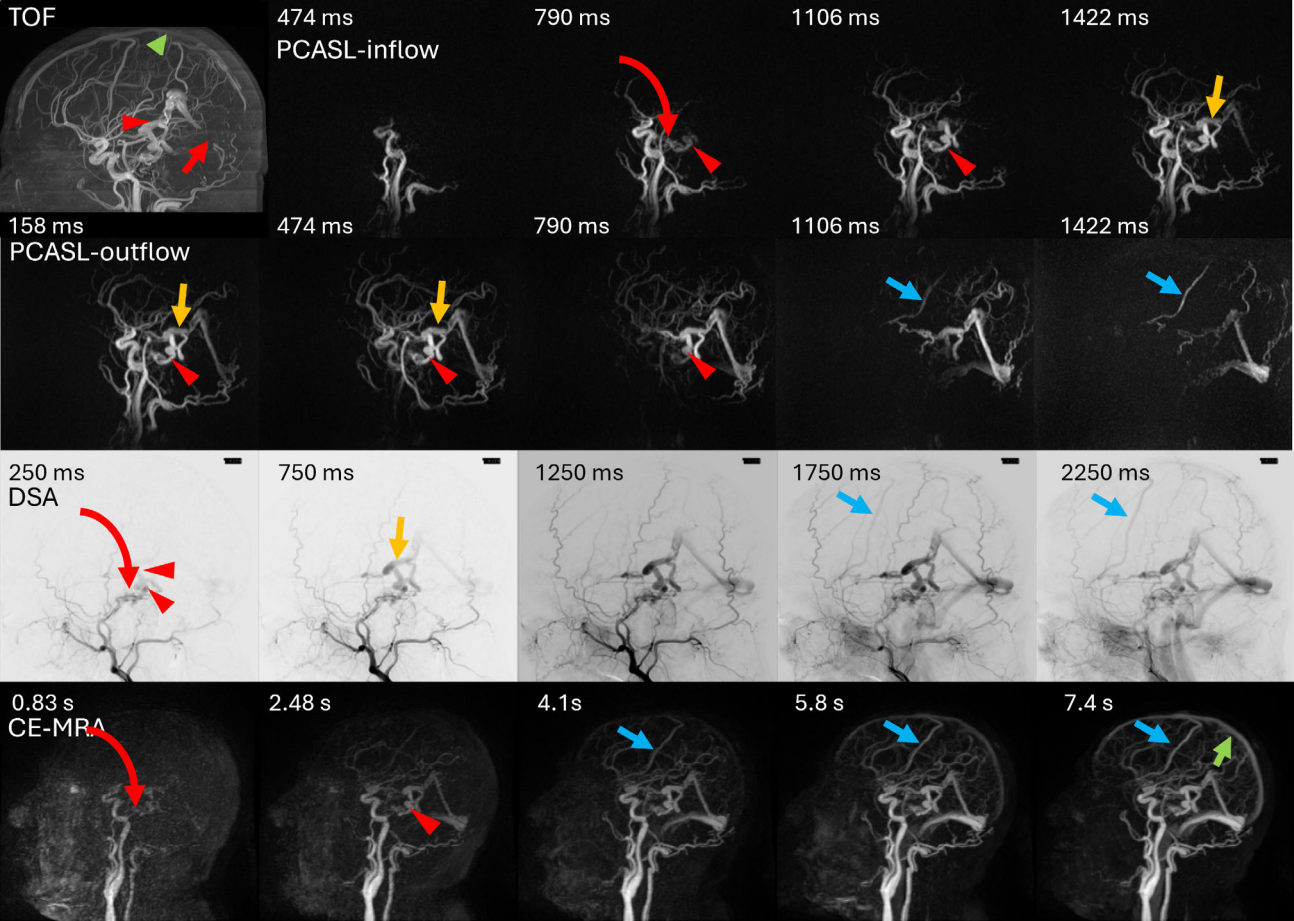
A 46‐year‐old man with a Cognard IV right tentorial dural arteriovenous fistulas (DAVF) (red curved arrow) draining via a dilated pontomesencephalic vein (red arrowhead) into the dilated right basal vein of Rosenthal (yellow arrow). Both arterial spin labeling (ASL) outflow and inflow subtraction images are shown. The tentorial shunt and the pontomesencephalic vein into which the fistula directly drains are better visualized on inflow images, on which downstream veins are only visualized at later timepoints, avoiding clutter. However, downstream veins, including reflux into the right Sylvian and superior anastomotic veins (blue arrows), are better visualized on the outflow images. This highlights a major advantage of ASL, only veins with shunted blood have a high signal. Conversely, even uninvolved veins opacify at later time points (green arrow) on contrast‐enhanced (CE)‐MRA, obscuring veins with delayed or smaller volume shunting that are only appreciable at later time points. The CE‐MRA also has much lower temporal resolution than pseudo‐continuous arterial spin labeling (PCASL) MRA. The entire ASL time course shown within the first 1106 ms is condensed into the two initial time frames of the CE‐MRA. Disadvantages of time of flight (TOF) MRA include spurious venous signal (green arrowhead) because of fast‐flowing blood or craniocaudal flow direction, leading to false positives, and suppression of blood signal (shown in straight sinus, red arrow) because of saturation effects, which may cause false negatives for low‐flow shunts. Please note that time points for digital subtraction angiography (DSA) are relative to the onset of direct arterial contrast injection into the external carotid artery, for PCASL MRA relative to labeling, and CE‐MRA relative to the start of intravenous injection, accounting for the differences at the same phase of arterial and venous opacification.

Figure 5 demonstrates dynamic PCASL MRA (both native outflow and inflow subtraction), dynamic CE‐MRA, DSA, and TOF MRA images in a patient with a Cognard IV DAVF. Although the spatial resolution of the two scans is comparable, the temporal resolution of PCASL MRA is substantially better than that of dynamic CE‐MRA. The earlier arterial phase ASL images demonstrate the tentorial shunt and pontomesencephalic vein into which it drains directly. This allows accurate determination of the drainage pattern (direct leptomeningeal vein drainage, hence Cognard IV). Conversely, even the earliest CE‐MRA image is later in the arterial phase, and signal is already seen in the straight sinus. This precludes accurate identification of the shunt point and drainage pattern. Later CE‐MRA phases demonstrate opacification of normal veins, such as normal cortical veins, which do not drain shunted blood. This results in a “cluttered” appearance with obscuration of draining veins of the DAVF with delayed or smaller volume shunting that is only appreciable at later time points. This highlights a major advantage of ASL: only veins with shunted blood have high signal. Comparison with catheter‐based cerebral DSA demonstrates that the draining veins are accurately captured on PCASL MRA, despite its lower spatial resolution. In fact, the PCASL MRA has superior temporal resolution to this DSA (200 vs. 500 ms). We have only shown the collapsed sagittal ASL images for each time point to make it comparable with DSA. However, the 3D acquisition allows reconstruction of the entire volume, which can be viewed as multi‐planar reconstruction to resolve fine detail and avoid overlapping vascular structures. The 2D planar projectional nature of DSA does not permit this. Finally, although 3D TOF MRA demonstrated higher spatial resolution than ASL, with better visualization of small and distal vessels, this sequence suffers from venous contamination because of incomplete suppression of venous signal in the cranial parts of the scan. Moreover, retrograde flow is suppressed either because of tracking saturation bands or RF saturation effects of the inflowing blood despite the use of ramped flip angle pulses. TOF also lacks temporal information, which prevents accurate determination of venous drainage patterns.

**FIGURE 6:**
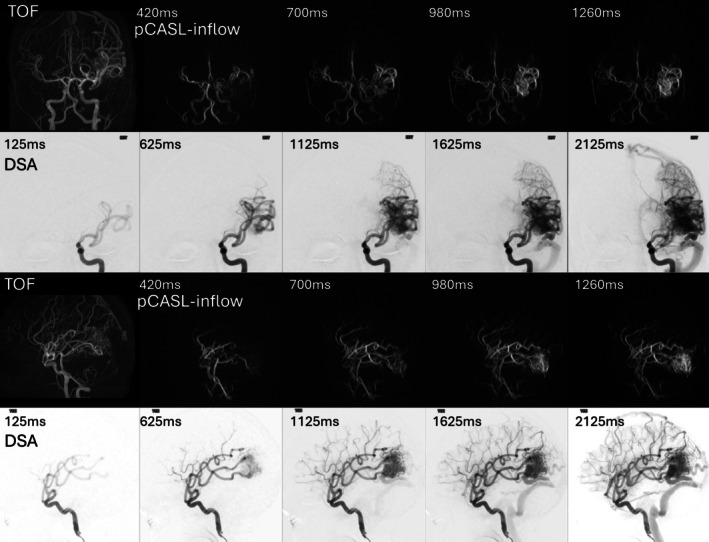
Time of flight (TOF), digital subtraction angiography (DSA), and pseudo‐continuous arterial spin labeling (PCASL) MRAs of a patient with a 28‐year‐old woman with left parietotemporal arteriovenous malformations (AVM), with superficial venous drainage into ectatic cortical veins, including the vein of Labbe. PCASL MRA was acquired with a matrix size of 256^3^ pixels. The temporal resolution of PCASL (280 ms) was comparable to DSA (250 ms) of which only every second frame is shown. Three enlarged left middle cerebral artery branches are seen to supply the nidus on both arterial spin labeling (ASL) and DSA, and the posterior cerebral artery  feeder is also well seen on ASL.

Figure 6 shows coronal and sagittal views of the PCASL MRA with inflow subtraction, TOF MRA, and DSA of a patient with a left parietotemporal AVM. Although the spatial resolution of PCASL MRA is lower than that of TOF and DSA, it still displays the relevant anatomical detail, including the three enlarged middle cerebral artery and posterior cerebral artery feeders, with a similar temporal resolution of 280 ms.

**FIGURE 7:**
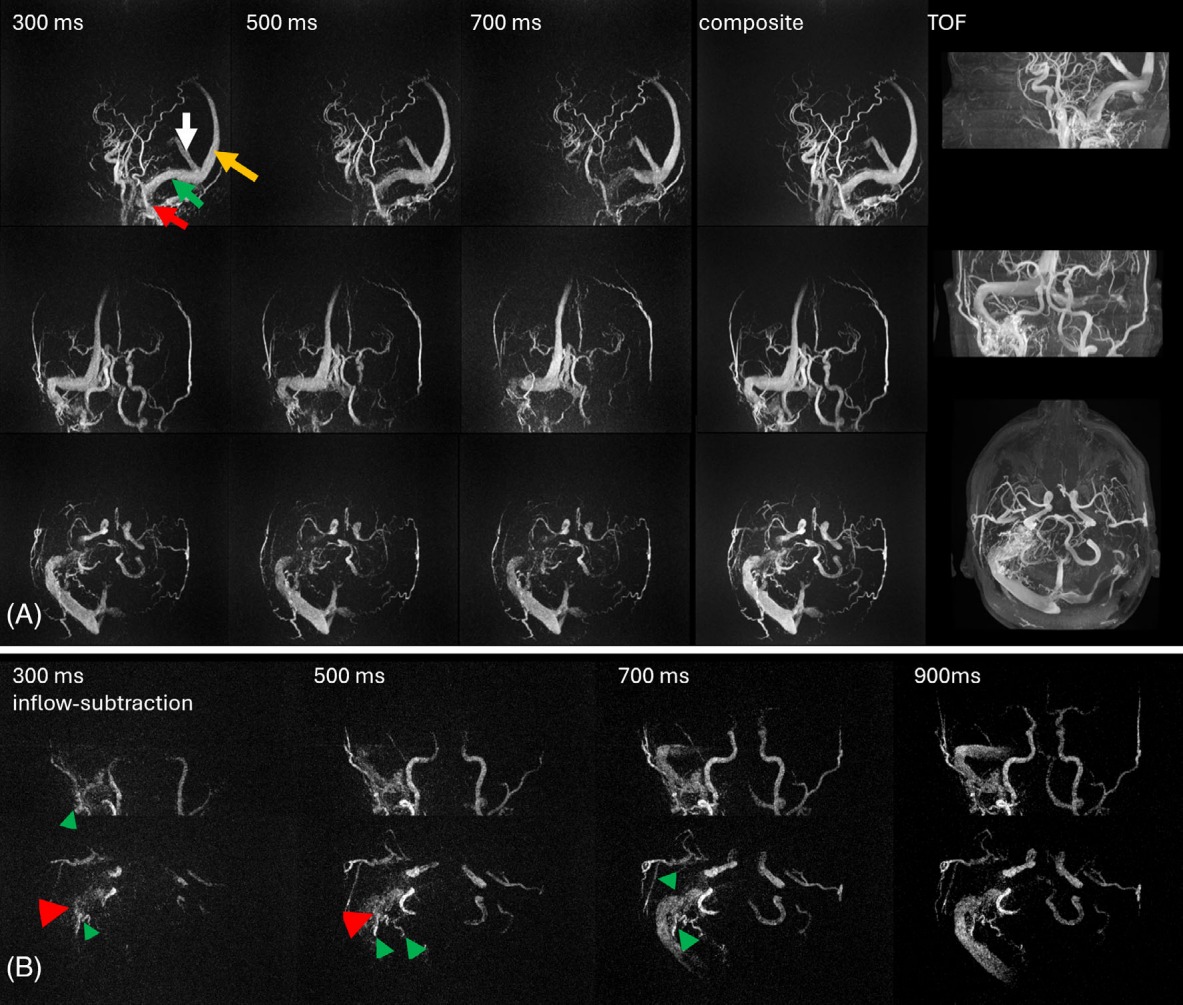
(A) Outflow images at three time points from a dynamic pseudo‐continuous arterial spin labeling (PCASL) MRA series, a composite reconstruction, and time of flight (TOF) images of Cognard 2A sigmoid sinus dural arteriovenous fistulas (DAVF) in a 42‐year‐old man who presented with blurred vision and other signs and symptoms of raised intracranial pressure. In this high‐flow shunt, outflow arterial spin labeling (ASL) images at 300 ms show extensive venous signal, within the sigmoid (red arrow), transverse (green arrow), superior sagittal (yellow arrow), and straight (white arrow) sinuses. The exact shunt point and sequence of venous drainage are, therefore, unclear. (B) The inflow images at different time points are much better for characterizing the shunt point and drainage pattern: signal is first seen in the sigmoid sinus shunt (red arrowhead), and subsequent images show signal in the transverse sinus because of reflux (signal later appears in the straight and superior sagittal sinuses, not shown here). The inflow images also demonstrate numerous feeding arteries arising from branches of the right external carotid artery (green arrowheads), visualized better than on TOF MRA, as there is no clutter from the draining veins at the early time points. The high resolution of 0.69 mm^3^ results in slightly lower SNR than the lower‐resolution ASL images shown in Figures 5 and 6. However, the composite reconstruction exhibits a strong signal and can be used as an anatomical reference for the time series.

Figure 7 shows another patient with a DAVF, highlighting the ability of PCASL MRA to achieve excellent spatial resolution (0.69 mm^3^) while simultaneously providing valuable dynamic information, in contrast to TOF. Because this patient had an allergy to MR contrast agents, and no CE‐MRA could be acquired, the ability of the ASL sequence to obtain dynamic information to determine the shunt point and venous drainage pattern accurately was particularly advantageous.

## 4 DISCUSSION

This study introduces a novel ASL angiography sequence that considerably enhances acquisition efficiency and aids in diagnosing cerebral vascular malformations. By leveraging the strengths of pseudo‐continuous ASL and fast, Look‐Locker‐type, dynamic multi‐spoke 3D‐radial SPGR, this method enables the detailed visualization of vascular structures without requiring exogenous contrast agents. Relative to CE‐MRA, this avoids the risk of adverse effects associated with contrast injections and the costs for contrast material and consumables, and simplifies workflows (no prior cannulation).

The multi‐spoke approach retains all the features of previously published 3D‐radial sequences,^16^, ^21^ while substantially reducing scan time. As a result, 3D PCASL MRA achieves faster acquisitions than 3D TOF MRA with similar spatial resolution and whole‐brain coverage, and the benefit of providing dynamic information, which allows delineation of venous drainage patterns. Despite these benefits, 3D PCASL MRA should be seen as a complement instead of a replacement for TOF MRA, because TOF MRA has become an extremely reliable and robust method for visualizing vascular anatomy. However, 3D PCASL MRA primarily focuses on collecting functional information and may, therefore, serve as an alternative to CE‐MRA.

Our experiments indicated that using up to three spokes yielded promising results within clinically feasible acquisition times. The results demonstrated similar image quality, superior SNR, and enhanced efficiency compared to single‐spoke scans. The improved SNR may be attributed to the increased TR and fewer RF pulses during the readout, reducing saturation effects and allowing for higher flip angles. Combined with an optimized flip angle progression, this approach maintained a stable signal with enhanced SNR across all temporal phases. Later phases, however, suffered from decreased overall image quality and increased artifact occurrences, the causes of which remain unclear and warrant further investigation. Motion‐related artifacts, potentially arising from elevated background signal because of the increasing flip angle schedule, might cause this behavior. Limited accuracy for T_1_ of blood and B_1_+ inhomogeneities can further exacerbate the degradation of later phases. Despite the excellent overall performance of the multi‐spoke approach, SNR gains are limited by signal loss because of T_2_* decay and off‐resonance effects, which may also explain the decreasing sharpness of multi‐spoke acquisitions. Additionally, because of longer TEs, flow‐related dephasing might cause signal loss in multi‐spoke acquisitions, as seen in the carotids in Figure 2.

With the proposed data acquisition scheme, an ultra‐short‐TE FID was also acquired at the start of each readout. This additional data further enhances SNR when included in the reconstruction. Furthermore, the acquired k‐space data can be combined to form a high‐SNR composite angiogram across all or a portion of the temporal frames, and a dynamic series with a high temporal resolution. Although Cartesian methods can apply view‐sharing and keyhole techniques,^14^ the radial trajectory is inherently more versatile and supports sliding‐window reconstructions.

The inflow subtraction technique effectively highlights the filling of the arterial tree, producing images that closely resemble CE‐MRA. However, robust subtraction across all temporal phases requires long ASL boluses and readouts. Inadequate arterial filling or incomplete drainage can lead to poor delineation of distal vessels.

As in CE‐MRA, the tissue background signal in PCASL MRA is effectively suppressed because of the label‐control subtraction. This contrasts with other inflow‐related techniques, such as TOF MRA or inflow inversion recovery, where the non‐vascular background is in some specific steady state or relaxing after an initial inversion, respectively.

Although we used coil channel compression and a gridding reconstruction, further improvements in image quality for the 3D‐radial PCASL MRA could be achieved through advanced reconstruction techniques, such as compressed sensing,^28^ temporal regularization,^28^, ^29^ or artificial intelligence‐based methods (including reconstruction, denoising, resolution enhancement^30^). These approaches potentially reduce acquisition time while maintaining or even enhancing image quality. At present, although reconstruction times for these methods remain substantial^31^ and were not used because they would be prohibitively long and a barrier to clinical adoption. This may be overcome in the future with faster hardware.

Off‐resonance effects constitute another factor influencing PCASL MRA image quality. On the one hand, PCASL labeling efficiency relies on a homogenous B_0_ field at the position of the labeling plane. On the other hand, image formation and spin dephasing are influenced by B_0_ inhomogeneities in the imaging volume. Hence, a tradeoff was chosen by enlarging the shimming volume to include both the label plane and imaging volume. Individual shimming of both the labeling and imaging planes and rapidly switching between these two shim settings, as suggested by Yang,^32^ was not performed. Furthermore, instead of magnitude addition, a dedicated off‐resonance reconstruction^33^ can prevent destructive interference in multi‐spoke acquisitions and further improve image quality.

Compared to previously published non‐Cartesian ASL MRA sequences, the proposed sequence generated images with considerably improved spatial and temporal resolution, albeit with a slightly longer acquisition duration. Wu et al.^16^ used a similar 3D‐radial PCASL sequence with variable label durations, achieving whole‐head coverage (0.68 mm^3^ isotropic) in 7:30 to 8:30 min, compared to 8:17 min scan time in this study. However, their temporal footprint of 500 ms is substantially longer than the 200 ms achieved here. In prior studies, we reported shorter imaging times of 4:52 min using a non‐Cartesian cone trajectory with a Look‐Locker‐type readout (Kopeinigg et al.^15^), although with a temporal footprint of 432 ms, an acquisition matrix of 200 × 200 × 75 pixels, and a non‐isotropic voxel volume that was six times larger, particularly with a fourfold increase in the caudo‐cranial direction. Other similar sequences using radial or cone trajectories^18^, ^29^, ^31^ focus on vessel‐selective ASL MRA or combine MRA and perfusion imaging, making direct comparison challenging.

Traditional methods, such as 3D TOF MRA and 3D CE‐MRA, are prone to (1) venous contamination (especially normal veins); (2) vessel saturation (TOF) effects (especially for in‐plane flow); (3) false luminal signal (T_1_‐shortening clot); and (4) retrograde venous flow entering a TOF slab proximally (Figure 7), complicating the assessment of shunting high‐flow lesions. Although tracking saturation bands were used for the TOF acquisitions, venous signal suppression was incomplete in the superior sagittal sinus (Figures 2 and 7), which may be because of suboptimal placement and width of the saturation band. Moreover, saturation was successfully applied to regions of retrograde flow in the patient (Figure 7), where it is, in fact, undesired.

In contrast, our ASL sequence distinctly images the arterial phase, which is particularly important for pinpointing the shunt location in the very early phase. This allows for clearer differentiation between arterial and venous structures when shunts are present, and tissue is bypassed. Three‐dimensional TOF MRA typically acquires multiple overlapping slabs to generate static angiograms within a scan time comparable to our sequence.

Three‐dimensional CE‐MRA exhibits substantially lower temporal resolution than our sequence and requires the administration of a contrast agent with the precise timing of the acquisition around the time of bolus arrival. This additional procedural step is also necessary for DSA, where separate injections are required in different arteries. Although DSA offers superior spatial resolution compared to ASL, its temporal resolution is comparable. Higher frame rates would come at the cost of moderately increased radiation exposure. Moreover, DSA is a projection technique lacking the multi‐planar reformatting capabilities available with the proposed sequence. A diagnostic DSA is also associated with small, but significant procedural risks. For patients with suspected, but not yet confirmed AVMs or DAVFs, for example, when referred to a service with pulsatile tinnitus, the use of time‐resolved PCASL MRA can be a valuable addition to such MR protocols.

## 5 CONCLUSION

A multi‐spoke 3D‐radial PCASL angiography sequence was developed, offering both high spatial and temporal resolution and sufficient SNR within a clinically acceptable scan time. This represents a significant step forward in non‐invasive, time‐resolved vascular imaging without the need for contrast agents. Despite the lower spatial resolution compared to DSA, the benefits of 3D PCASL MRA are substantial, including comparable spatial resolution to TOF MRA, avoidance of ionizing radiation, procedural complications, and costs associated with DSA. In addition, specialized interventional neuroradiology services for cerebral DSA are not as widely available as MRI scanners, particularly in smaller and community hospitals. Therefore, this MRI technique for detecting and characterizing high‐flow vascular lesions improves access to diagnosis for patients.

## Supporting information


**Figure S1.** Comparison of the optimized flip angle evolution for the 1 to 3‐spoke acquisitions. Note that the larger TR and lower number of excitation pulses in the 3‐spoke acquisitions allow for a higher flip angle throughout the progression compared to the other scans.
**Figure S2.** Comparison of axial MIPs of a dynamic pseudo‐continuous ASL (PCASL) MRA series produced with 1–3 spokes and inflow subtraction in a 35‐year‐old healthy volunteer. All temporal frames demonstrate high quality and detail, with progressive arterial filling visible throughout the time series.
**Figure S3.** Example of artifacts, usually occurring in later temporal phases.

**Video S1.** Schematic video of k‐space traversal.

**Video S2.** Outflow video created from a sliding‐window reconstruction of volunteer one.

**Video S3.** Inflow video created from a sliding‐window reconstruction of volunteer one.

**Video S4.** Outflow video created from a sliding‐window reconstruction of patient three.

**Video S5.** Inflow video created from a sliding‐window reconstruction of patient three.

**Video S6.** Outflow video created from a sliding‐window reconstruction of patient six.

**Video S7.** Inflow video created from a sliding‐window reconstruction of patient six.
