# Supplementary material for: Dynamic pseudo‐continuous arterial spin labeling angiography using a 3D‐radial multi‐spoke spoiled gradient‐recalled sequence
Source: Magn Reson Med. 2025 Sep 17;95(2):724–39. doi: 10.1002/mrm.70015 (PMC12681305; doi:10.1002/mrm.70015)
Supplement: Supplementary file 1 — Figure S1. Comparison of the optimized flip angle evolution for the 1 to 3‐spoke acquisitions. Note that the larger TR and lower number of excitation pulses in the 3‐spoke acquisitions allow for a higher flip angle throughout the progression compared to the other scans. Figure S2. Comparison of axial MIPs of a dynamic pseudo‐continuous ASL (PCASL) MRA series produced with 1–3 spokes and inflow subtraction in a 35‐year‐old healthy volunteer. All temporal frames demonstrate high quality and detail, with progressive arterial filling visible throughout the time series. Figure S3. Example of artifacts, usually occurring in later temporal phases. [file MRM-95-724-s005.docx]

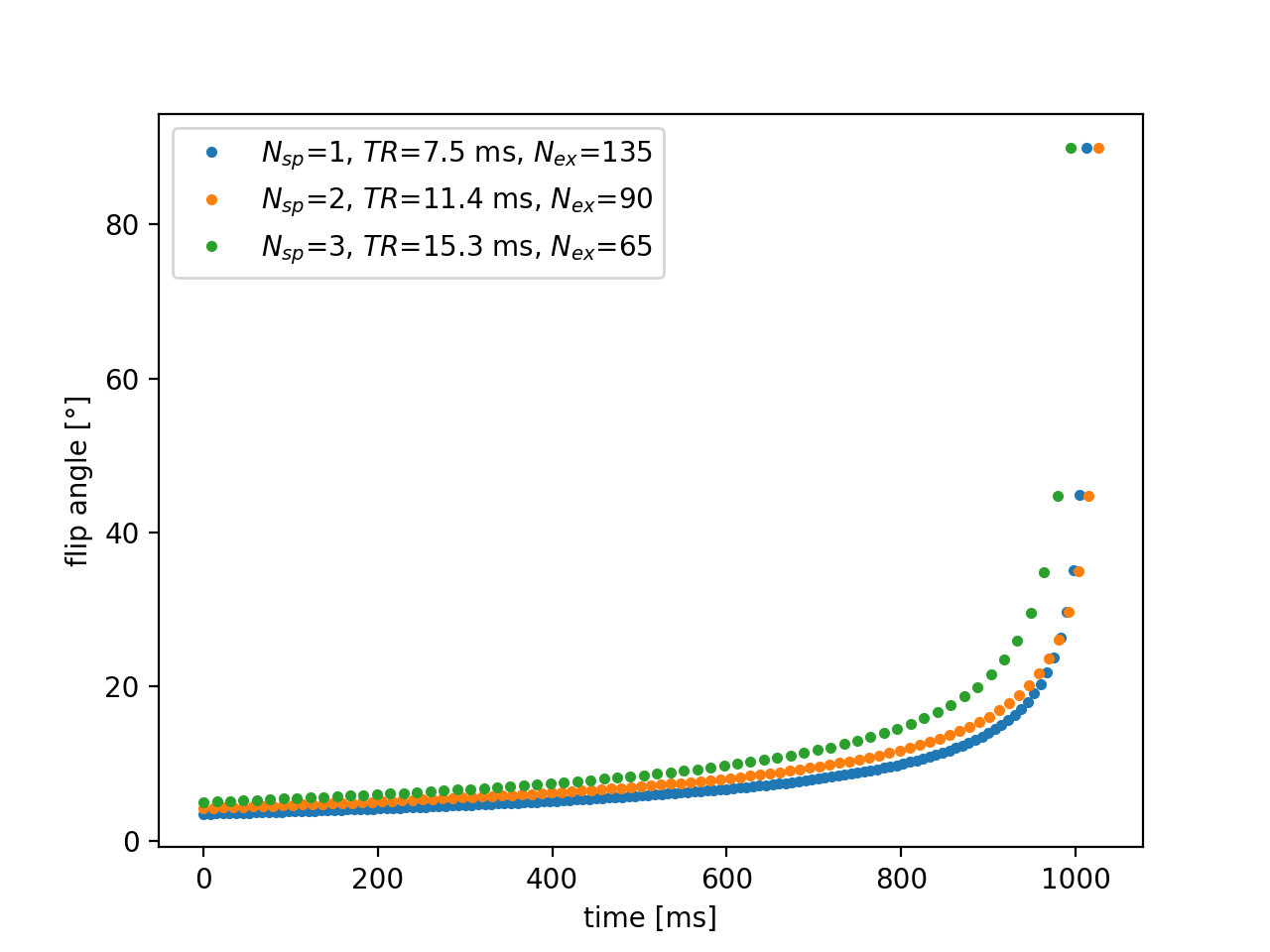


Figure S1: Comparison of the optimized flip angle evolution for the 1 to 3-spoke acquisitions. Note that the larger TR and lower number of excitation pulses in the 3-spoke acquisitions allow for a higher flip angle throughout the progression compared to the other scans.


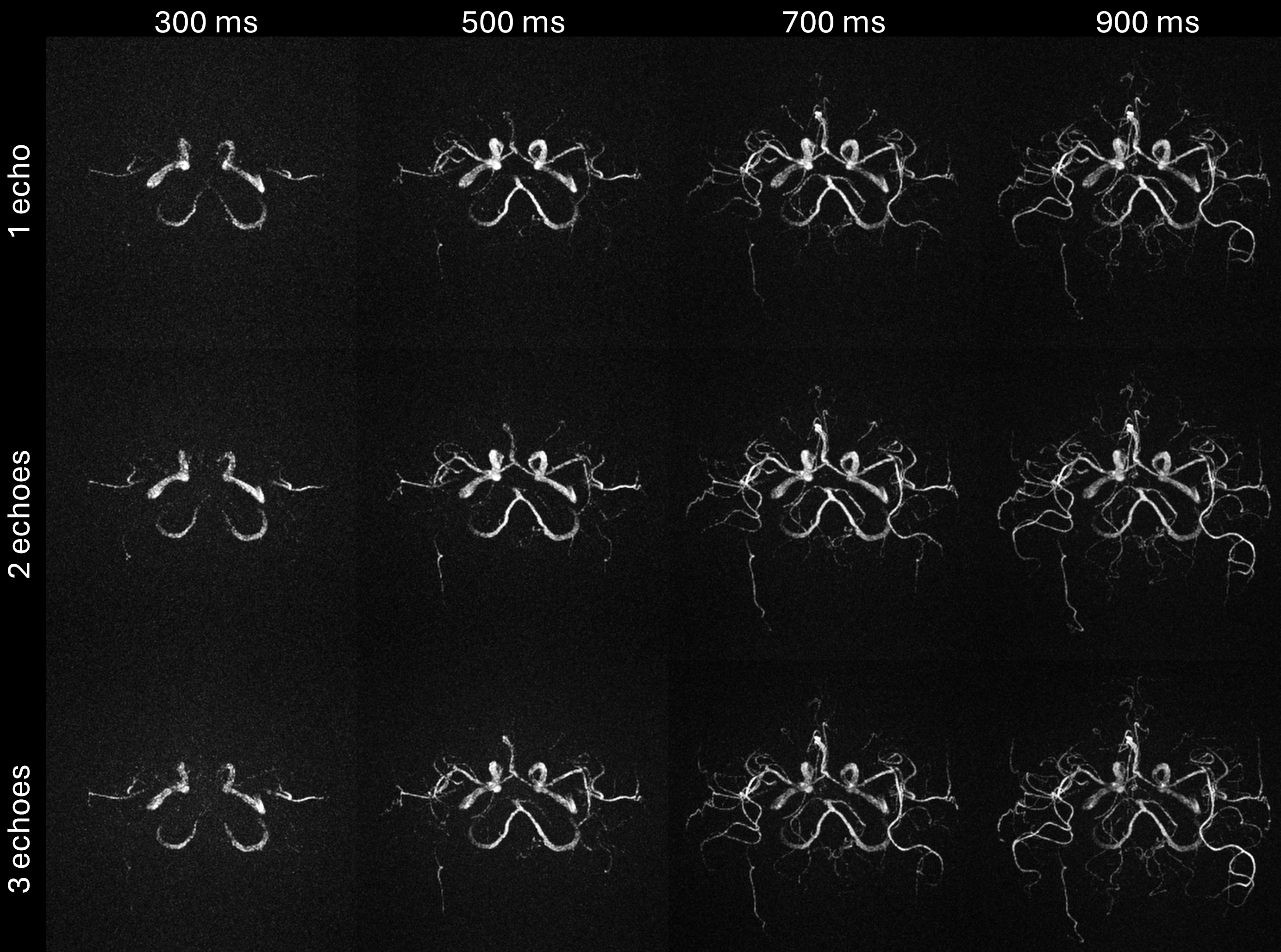


Figure S2:  Comparison of axial MIPs of a dynamic PCASL MRA series produced with 1-3 echoes and inflow-subtraction in a 35-year-old healthy volunteer. All temporal frames demonstrate high quality and detail, with progressive arterial filling visible throughout the time series.


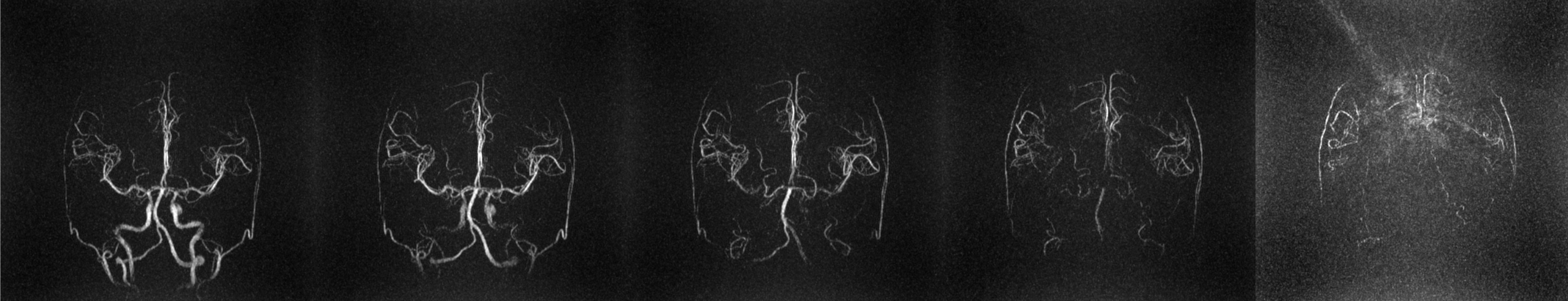


Figure S3: Example of artifacts, usually occurring in later temporal phases.
